# Supplementary material for: Investigation of Tumor Heterogeneity Using Integrated Single-Cell RNA Sequence Analysis to Focus on Genes Related to Breast Cancer-, EMT-, CSC-, and Metastasis-Related Markers in Patients with HER2-Positive Breast Cancer
Source: Cells. 2023 Sep 15;12(18):2286. doi: 10.3390/cells12182286 (PMC10527746; doi:10.3390/cells12182286)

**Shiino S et al.**

## Figure S1. Single-cell analysis of the epithelial components of luminal-HER2 (ER-positive/HER2-positive) breast cancer

(A) A UMAP plot of epithelial cells in the luminal-HER2 breast samples.
(B) UMAP plots with representative marker expression.
(C) Violin plots with representative marker expression.

A

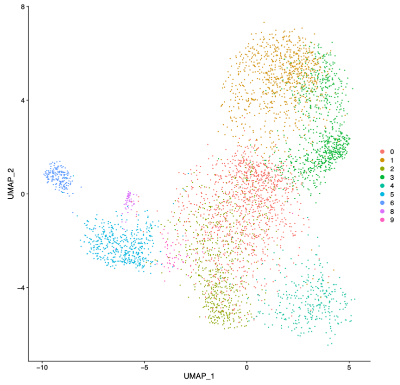


B

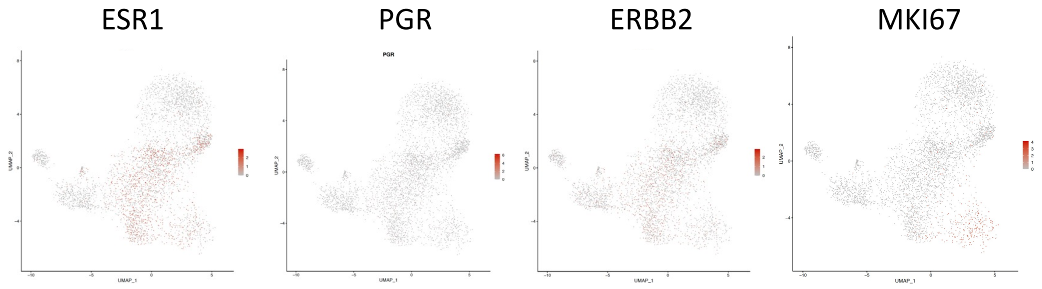


C

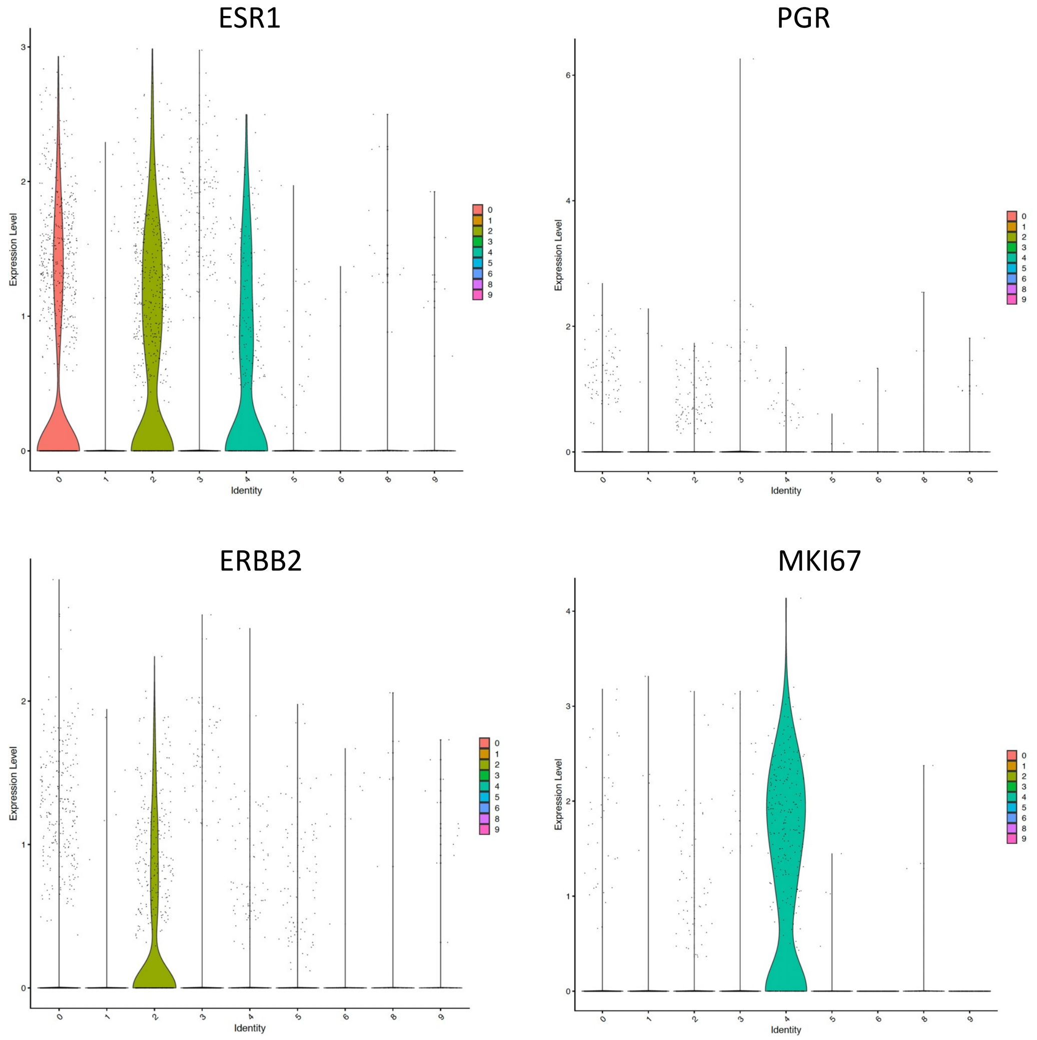

**Shiino S et al.**

A

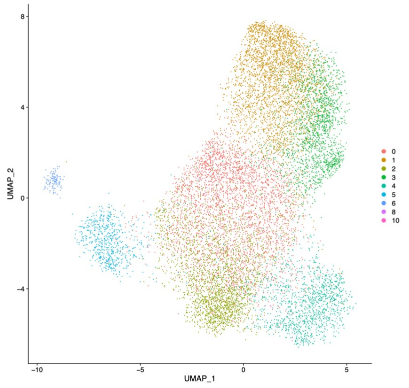


**Figure S2. Single-cell analysis of the epithelial components of pure-HER2 (ER-negative/HER2-positive) breast cancer**

(A) A UMAP plot of epithelial cells of the pure-HER2 breast samples.

(B) UMAP plots with representative marker expression.

(C) Violin plots with representative marker expression.

B

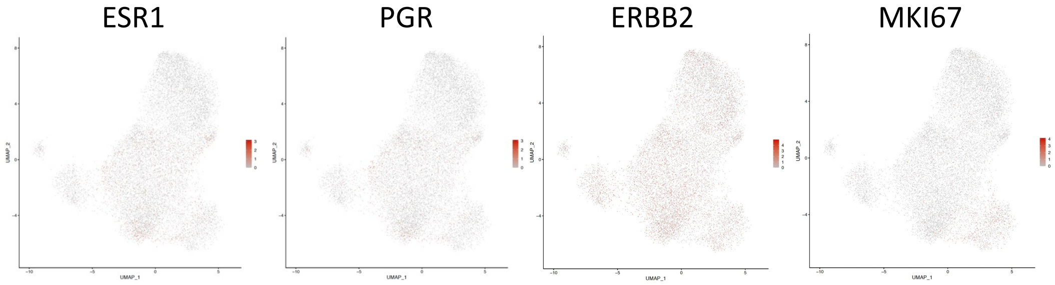


C

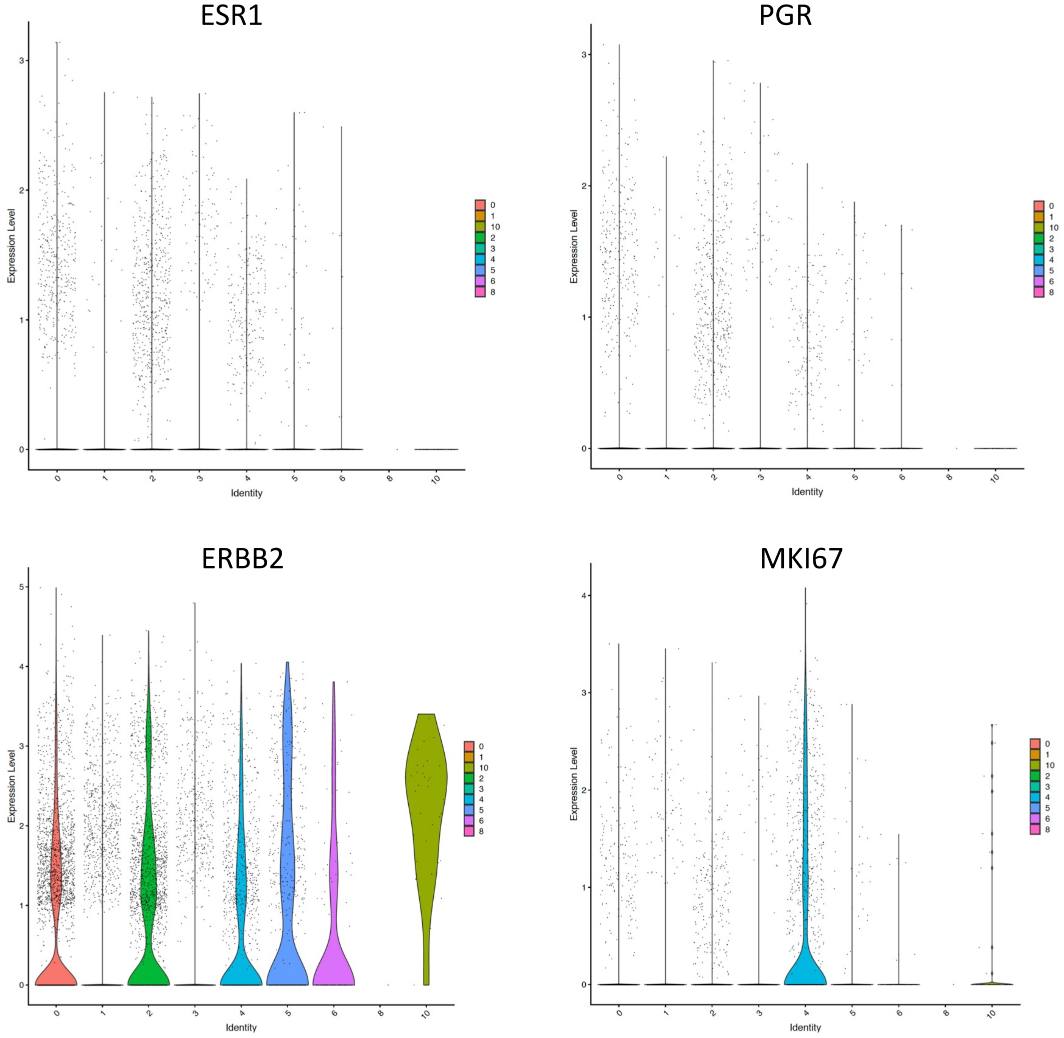

Shiino S et al.

A

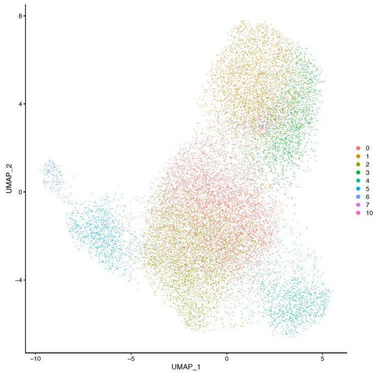


**Figure S3. Single-cell analysis of the epithelial components of luminal (ER-positive/HER2-negative) breast cancer**

(A) A UMAP plot of epithelial cells of the luminal breast samples.
(B) UMAP plots with representative marker expression.
(C) Violin plots with representative marker expression.

B

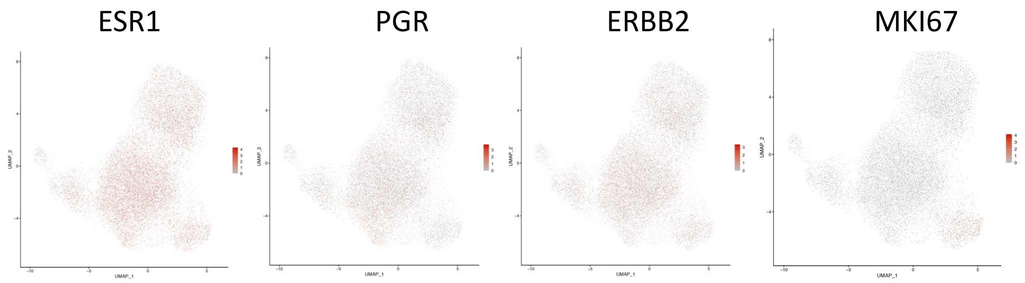


C

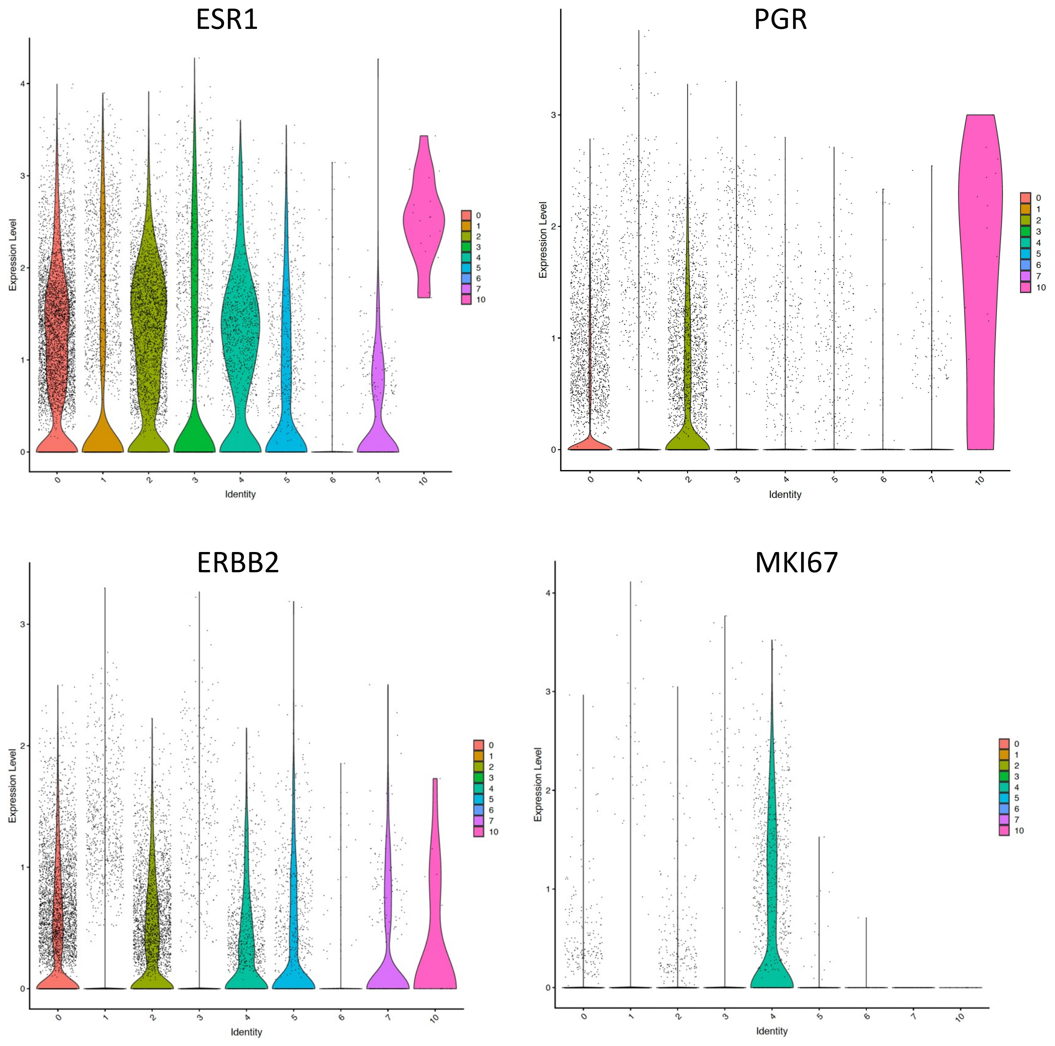

**Figure S4. Comparison of gene expression levels of representative breast cancer markers among the Luminal-HER2 subtype, pure-HER2 subtype, and Luminal subtype**

H: Luminal-HER2 subtype, L: pure-HER2 subtype, Luminal: Luminal subtype.
N: The presence of HER2 amplification but not available for hormone receptor status on IHC results (Details are shown as Table S1 (a row named “Subtype)).

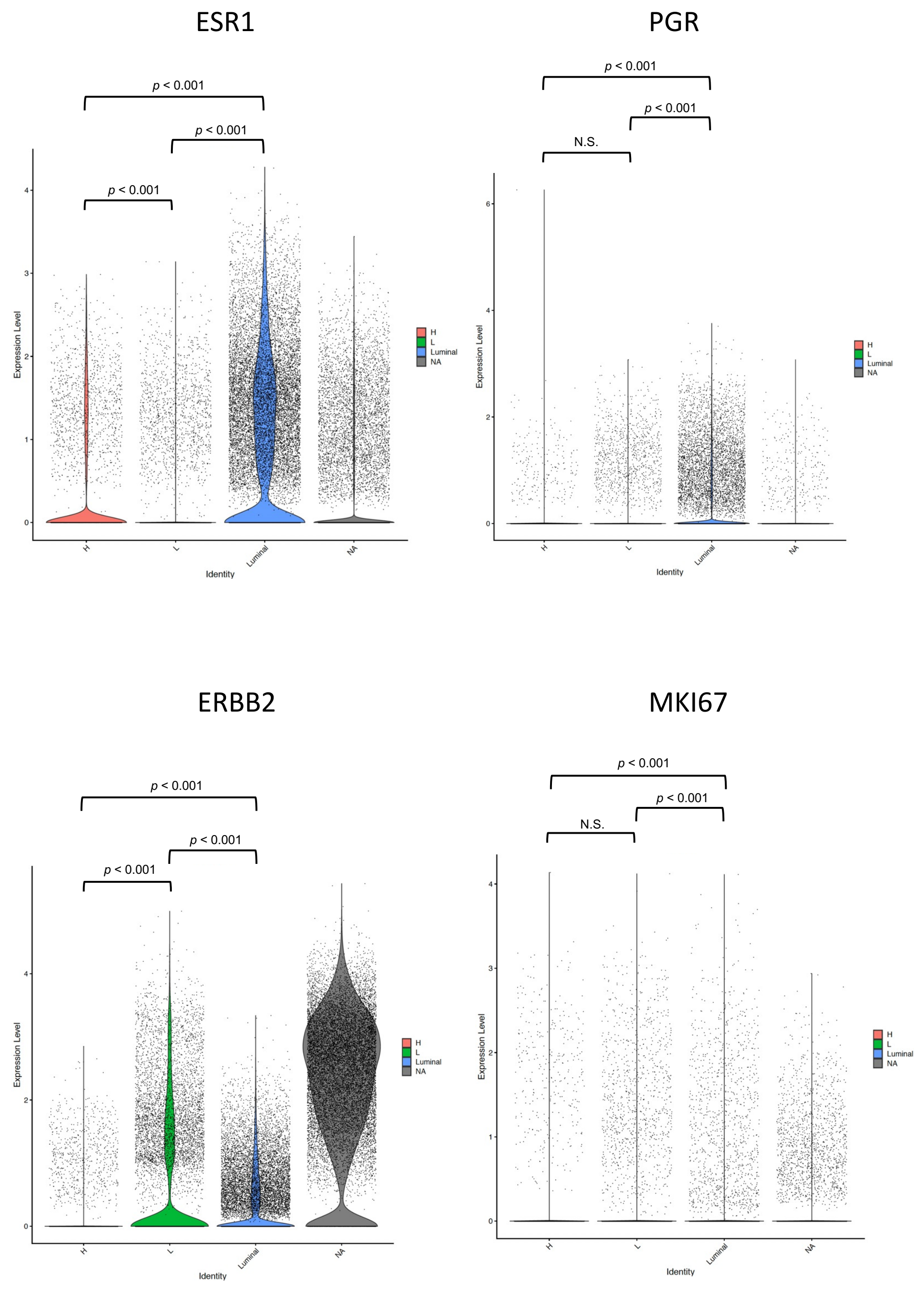

Supplement: Supplementary file 1 [file cells-12-02286-s001.zip › Supplementary Figure.pdf]
